# Field diagnosis and genotyping of chikungunya virus using a dried reverse transcription loop-mediated isothermal amplification (LAMP) assay and MinION sequencing

**DOI:** 10.1371/journal.pntd.0007480

**Published:** 2019-06-03

**Authors:** Kyoko Hayashida, Yasuko Orba, Patricia C. Sequeira, Chihiro Sugimoto, William W. Hall, Yuki Eshita, Yutaka Suzuki, Lucky Runtuwene, Patricia Brasil, Guilherme Calvet, Cintia D. S. Rodrigues, Carolina C. dos Santos, Maria A. M. Mares-Guia, Junya Yamagishi, Ana M. B. de Filippis, Hirofumi Sawa

**Affiliations:** 1 Research Center for Zoonosis Control, Hokkaido University, Sapporo, Japan; 2 Flavivirus Laboratory, Oswaldo Cruz Institute/ Oswaldo Cruz Foundation (Fiocruz), Rio de Janeiro, Brazil; 3 Global Station for Zoonosis Control, Global Institution for Collaborative Research and Education (GI-CoRE), Hokkaido University, Sapporo, Japan; 4 Centre for Research in Infectious Diseases, School of Medicine and Medical Science, University College Dublin, Dublin, Ireland; 5 Global Virus Network, Baltimore, Maryland, United States of America; 6 Graduate School of Frontier Sciences, the University of Tokyo, Kashiwa, Japan; 7 Acute Febrile Illnesses Laboratory, Evandro Chagas National Institute of Infectious Diseases, Oswaldo Cruz Foundation (Fiocruz), Rio de Janeiro, Brazil; Faculty of Science, Mahidol University, THAILAND

## Abstract

Detection and sequencing of chikungunya virus (CHIKV) genome was performed using a combination of a modified reverse transcription loop-mediated isothermal amplification (RT-LAMP) method and a MinION sequencer. We developed the protocol for drying all the reagents for the RT-LAMP in a single reaction tube. Using this system, the CHIKV genome was effectively amplified under isothermal conditions, and used as a template for MinION sequencing with a laptop computer. Our *in-house* RT-LAMP method and MinION sequencing system were also validated with RNAs and serum samples from recent outbreaks of CHIKV patients in Brazil. The obtained sequence data confirmed the CHIKV outbreaks and identified the genotype. In summary, our established inexpensive on-site genome detection and sequencing system is applicable for both diagnosis of CHIKV infected patients and genotyping of the CHIKV virus in future outbreak in remote areas.

## Author summary

Chikungunya virus has re-emerged as an important pathogen causing several outbreaks in the world. As the clinical symptoms of chikungunya is similar to other mosquito-borne febrile diseases, the definitive diagnosis of the disease is based on the detection of viral genome from the patient blood. Loop-mediated isothermal amplification (LAMP) is a method that rapidly amplify nucleic acids under isothermal condition. In the present work, a simple dried format LAMP test for chikungunya diagnosis was developed which can be directly amplified from human blood. Combining with the portable sequencer MinION sequencing system, a method to identify the viral genotype was also established. The developed on-site diagnosis and genotyping system is easy to perform, sensitive, and rapid. Therefore, it offers great promise as a routine simple tool for diagnosis and disease management of chikungunya.

## Introduction

Chikungunya is a mosquito-borne febrile disease caused by chikungunya virus (CHIKV), which is a positive strand RNA virus belonging to the genus *Alphavirus*. In the last decade, serious outbreaks of chikungunya have been reported. These appeared to have started on the coast of Kenya in 2004 [1], and subsequently spread to islands in Indian ocean [2], India [3], parts of Southeast Asia [4], as well as temperate Mediterranean areas of Europe [5–7]. Since late 2013, chikungunya outbreaks have been also reported in the Caribbean, United States, Mexico, Central America, and Brazil [8–11]. Millions of people have been affected during these outbreaks, and infection is now a major public health concern [11–13].

CHIKV are phylogenetically classified into three major genotypes, the West Africa, the Asia, and the East-Central South Africa (ECSA). The Asian genotype was the cause of recent outbreaks in the Caribbean and United States [14]. In Brazil, both Asian and ECSA genotypes have been reported, with the outbreak in Rio de Janeiro beginning in 2014, being attributed to the ECSA genotype [9, 15, 16]. It has been reported that phylogenetic diversification and infectivity differences among CHIKV may be correlated [17] and the evolution of CHIKV may be related to the viral adaptation in mosquito vectors and mammalian hosts.

Since the clinical features of chikungunya are similar to other mosquito-borne febrile diseases, including Dengue fever, Zika fever and malaria, the differential diagnosis of these diseases based on clinical signs is difficult in endemic regions. Definitive diagnosis of chikungunya is based on serology, virus isolation, or genome detection by reverse transcription PCR (RT-PCR) from patient-derived samples. However, these laboratory diagnostic techniques are expensive and require well-equipped facilities that are not generally available in remote areas and thus are seldom performed in routine clinical practice.

Loop-mediated isothermal amplification (LAMP) is a very sensitive,“user-friendly”, and time-efficient nucleic acid amplification method [18, 19]. The method can be applied to detect genomes of RNA viruses if reverse transcriptase is included in the reaction (reverse transcription LAMP; RT-LAMP). Using the LAMP and RT-LAMP methods, amplification reaction of nucleic acids can be performed under isothermal conditions without expensive or sophisticated equipment. The *Bst* polymerase, a necessary enzyme for the LAMP reaction, is known to be highly tolerant to inhibitory molecules in clinical samples [20], and therefore it is applicable for the samples without the requirement of the purification steps of nucleic acids. We have already found that all the reagents for the LAMP can be dried and kept in reaction tubes, and that patient blood can be used directly in the LAMP reaction without purification of nucleic acids. This simplified protocol has been applied for diagnosis of Human African Trypanosomiasis and Malaria [21, 22]. This dried-LAMP (named CZC-LAMP) can be stored at the ambient temperature for prolonged periods, which is useful in the remote areas where cold-chains are not available.

Recently, a portable-type next generation sequencer, the MinION has been developed. The sample preparation from DNA or cDNA can be completed within 10 min in the simplest protocol. The library preparation for sequencing can be performed with a magnet separator and simple isothermal incubator, and thus only minimal equipment is required. Notably as most of the newly emerging infectious disease outbreaks have been reported in the remote areas with resource-poor settings, portable, affordable and disposable MinION provides a promising tool for rapid identification and epidemiological analysis on site.

In this study, we combined our field-friendly RT-LAMP system and the MinION technology, to successfully achieve viral sequencing in a simple and rapid way. Specifically, the genomes of CHIKV in a drop of human blood would be amplified with the dried RT-LAMP method, and the products sequenced by the MinION. This simple sequencing work-flow is likely to be applicable to investigate outbreaks of various infectious diseases in remote areas.

## Materials and methods

### Virus samples

Chikungunya virus SL11131 (AB455493) and SL10571 (AB455494), which are members of the ECSA genotype, were passaged in Vero cells. These viruses were isolated from serum of a Japanese patient returning from Sri Lanka in 2006 [23], who provided written informed consent for their use. CHIKV-S27 which is an African prototype (NC_004162) also belongs to the ECSA genotype. These viruses were provided by Dr. Takasaki (National Institute of Infectious Diseases, Japan) and stored in -80 ^o^C freezer at a biosafety level (BSL)-3 until use.

### Preparation of in-house dried CHIKV RT-LAMP (CHIKV-CZC-LAMP) system

The in-house dried CHIKV RT-LAMP system was produced by using a trehalose vitrification technique based on a previous report [21] with several modifications. Trehalose (FUJIFILM Wako Pure Chemical, Osaka, Japan) was prepared by dissolving in deionized distilled water (2 mol/L = 2M) in 85 ^o^C for 1 hour. The Trehalose solution (1.6 μl, 2M), deoxyribonucleotide triphosphates (dNTPs) (1.4 μl, 25mM each) (Nippon Gene, Tokyo, Japan), WarmStart RTx reverse transcriptase (0.25 μl, 15 U/μl) (New England Biolabs Inc., Ipswich, MA), RNase inhibitor (0.1 μl, 40 U/μl) (Takara Bio Inc., Shiga, Japan), and *Bst* 2.0 WarmStart DNA polymerase (0.05 μl, 120 U/μl and 0.25 μl, 8 U/μl) (New England Biolabs Inc.) were then mixed. We used two different concentrations of *Bst* 2.0 WarmStart DNA polymerase to adjust the glycerol amount in the reaction mixtures, which resulted in an effective drying time and enzyme stability [21]. The enzyme mixture solution (3.65 μl) was placed inside of the tube lid. The LAMP primer sets for CHIKV [24] were prepared in deionized distilled water. FIP and BIP (0.4 μl each), F3 and B3 (0.05 μl each), and FLF and BLP (0.2 μl each), trehalose (0.7 μl, 2M), and the colori-fluorometric indicator (CFI)(1 μl) were mixed and the mixture solution (2.35 μl) was placed at the bottom of the same reaction tube as the enzyme mixture solution had been placed on the lid. CFI consists of 3 mM hydroxyl-naphtol blue (HNB; MP Biomedicals, Aurora, OH) and 0.35% v/v GelGreen (10,000X solution in DMSO, Biotium, Hayward, CA) dissolved in distilled water. The tubes were air dried with a fan in a grove box connected with an ultra-low dew point air dryer (QD20-50; IAC Co., Kawasaki, Japan) for 12 hours. The tubes with dried mixture solutions were kept with molecular sieves 3A (FUJIFILM Wako Pure Chemical) in an aluminium bag at ambient temperature.

### Detection of CHIKV genome in whole blood using the CHIKV-CZC-LAMP system

The reaction tubes with the dried mixture solutions were stored for at least 2 months at ambient temperatures and were emplaced in the RT-LAMP reaction for CHIKV genome detection. Prior to the reaction, reaction buffer (23 μl), consisting of 20 mM Tris-HCl (pH8.8), 50 mM KCl, 6 mM MgSO_4_, and 10 mM (NH_4_)_2_SO_4_ in 0.1% TritonX-100, and 2 μl template was added. For the templates, 1 μl of extracted RNA with or without 1 μl of whole blood from healthy human volunteer were used. RNAs were extracted from CHIKV when titers were determined by plaque forming units per ml (PFU/ml). Thereafter, the tubes were turned upside down for 2 minutes to mix and reconstitute the dried enzyme reagents. The RT-LAMP reaction was achieved at 63 ^o^C for 45 minutes. Because CHIKV-CZC-LAMP contains gelgreen which emits green fluorescent, the reaction could be monitored by the FAM channel with real-time PCR detection system (CFX96; Bio-Rad, Philadelphia, PA). One cycle of the amplification was set as 1 min, and reaction speed (min) was estimated to be equal to the threshold cycle (Ct) value. The specificity was judged by Tm (melting temperature) value.

### Detection of CHIKV genome from clinical samples using CHIKV-CZC-LAMP

Our assay was also validated using clinical samples. Serum samples were obtained from clinically suspected patients collected in 2016 and 2018, and which had been stored at the Flavivirus Laboratory, Oswaldo Cruz Institute/Oswaldo Cruz Foundation (Fiocruz) in Rio de Janeiro which is a Brazilian Ministry of Health Regional Reference Laboratory for arboviruses (LABFLA).Viral RNAs from those sera were extracted by the QIAamp viral RNA mini kit (Qiagen, Germany). Detection of the genome of CHIKV by the CHIKV-CZC-LAMP was performed using directly the serum or RNA from sera samples (2 μl) with 23 μl of the reaction buffer in each tube. For incubation of the samples, the portable incubator (BSR-miniT100H, Bio Medical Science, Tokyo, Japan) was used, and detection of the fluorescent signal from reactive samples was confirmed using the hand-made blue-green LAMP reaction detector as described in our previous report [21]. To compare the sensitivity of the RT-LAMP system, quantitative real-time PCR (qRT-PCR) [25] was conducted with the Express One-step SuperScript qRT-PCR system (Invitrogen, Carlsbad, CA) and the StepOnePlus Realtime-PCR System (Thermo Fisher Scientific, Waltham, MA). The reaction cycle was set as 50 ^o^C for 15 minutes for reverse transcription, 95°C for 2 minutes for initial denaturation, followed by 45 cycles of 95°C for 15 seconds, and 60°C for 60seconds. The sequences of primer sets for the qRT-PCR were as follows; Forward primer 6856F: 5’-TCACTCCCTGTTGGACTTGATAGA-3’, reverse primer 6981R: F: 5’-TTGACGAACAGAGTTAGGAACATACC -3’, and 6919 FAM-MGB probe: 5’-FAM-AGGTACGCGCTTCAAGTTCGGCG -MGV-3’ [25].

### MinION sequencing using LAMP products

The genomic DNA Sequencing kit SQK-MAP-006 (Oxford Nanopore Technologies, Oxford Science Park, UK) was initially used for CHIKV SL10571 and S27 in which one sample was analyzed in an individual flow-cell. Briefly, LAMP products (2 μl) were directly used for end-repairing and dA-tailing using the NEBNext Ultra II End Repair/dA-tailing module (New England Biolabs) by incubation at 20°C for 5 minutes, then 65 ^o^C for 5 minutes, and subsequently purified by Agencourt AMPure XP beads (Beckman Coulter, Brea, CA) with a magnetic stand. Adaptors (Oxford Nanopore Technologies) were then ligated at room temperature for 10 minutes by Blunt/TA Ligase Master Mix (New England Biolabs). The resultant adapter-ligated DNA was purified by Dynabeads MyOne Streptavidin C1 (Thermo Fisher Scientific). Thereafter, samples were eluted with elution buffer (Oxford Nanopore Technologies) and the resultant libraries were applied to the MinION Flow-Cell (R7.0) with buffer and fuel mix, and the Genomic DNA 48-hour sequencing protocol was used with the MinKNOW software (Oxford Nanopore Technology). The base call was performed with the Metrichor Agent (https://metrichor.com) and only “passed” sequences with high reliability were used for the subsequent analysis. As for LAMP products from clinical samples, a 1D Native barcoding genomic DNA kit (with EXP-NBD103 and SQK-LSK108, Oxford Nanopore Technologies) was applied according to the manufacture’s instruction. Briefly, LAMP products (2 μl) were ligated with each barcode by Blunt/TA Ligase Master Mix (New England Biolabs), purified by AMPure XP beads, then all samples were pooled and applied to the single MinION Flow-Cell (R9.4, FLO-MIN106) in MK1b device.

### Sequence data analyses

The obtained FAST5 data from MinION were converted into fastq file formats using albacore.1.2.6 (Oxford Nanopore Technologies). MinION sequence data have been submitted to the DNA Data Bank of Japan (DDBJ) under the following DDBJ Sequence Read Archive (DRA) accession number: DRA007513. The local BLASTN program was employed with obtained reads as queries against CHIKV SL11131 (AB455493) reference at position of 10,317–10,472, which corresponds to the region between F2 to B2 region of LAMP reaction (E value <1e-5, identity >90%). All the blastn hit regions were clipped and aligned to CHIKV SL11131 by Bowtie2 [26], and IGV and IGVtools [27] were used to visualize and obtain count data from bam files. The obtained count data were analyzed by MicroSoft Excel (S1 Table), and consensus sequences were constructed. For further sequence analysis, the F2 and B2 regions were omitted, since during LAMP amplification those sequences would be replaced by the primer sequence completely. For the remaining 117 bp region (10,336–10,452), the population for the major nucleotides was interpreted as a consensus of the sequence, if the proportion of the main allele was supported by more than 70% of the total coverage. For bi-allelic loci, a non-primer allele was considered as a genuine allele taking into account for the primer effect, that partially replaced the original mutation into the primer sequence (LF/LB and F1/B1). The consensus sequences were aligned, and phylogenetic trees were constructed by a Neighbor joining (NJ) -based method with the reference CHIKV sequences [11, 16, 28, 29] using MEGA7 software [30].

### Ethics statement

Ethical approval for the diagnostic procedure was obtained from both the Ethical Screening Committee of Fiocruz (CAAE): 90249218.6.1001.5248 (2.998.362), and the Graduate School of Veterinary Medicine/ the Research Center for Zoonosis Control, Hokkaido University (approved number: 28–2). Because patient data was anonymized, informed consent was not required. Also, there is a transference agreement of LAMP and MinION protocols between Hokkaido University and Flavivirus Laboratory to support the virological surveillance performed by the LABFA, and all validation test using Brazilian sample were performed at this laboratory.

## Results

### Detection of CHIKV genome in blood samples using dried RT-LAMP system

**Fig 1:**
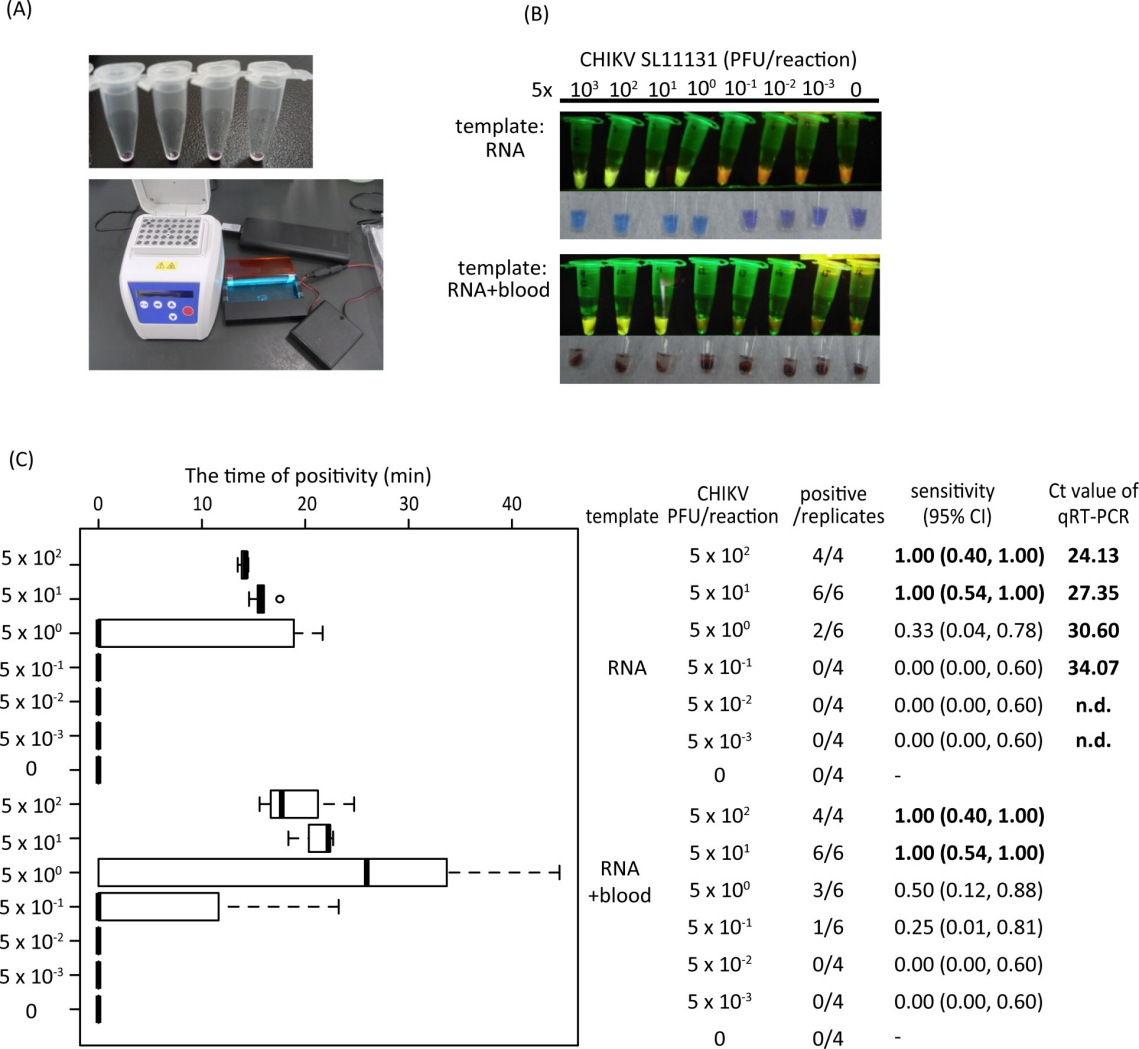
CHIKV-CZC-LAMP detected <50 PFU CHIKV. (A) CHIKV-CZC-LAMP reaction tubes (upper column) and the equipment used for the CHIKV-CZC-LAMP reaction and detection (lower column). CHIKV-CZC-LAMP reaction tubes have all-in-one dried reagents that can be stored at room temperature. The amplifications were performed at 63°C, and the positive yellow fluorescent signals were observed with the LED LAMP reaction detector. (B) Representative view of CHIKV-CZC-LAMP results. The decreasing concentration of CHIKV in 1 μl of RNA or 1 μl RNA spiked with 1 μl of healthy human blood were tested. As a negative control, blood without CHIKV RNA was used. Upper panel shows fluorescent signals detected by 505 nm blue green LED detector. Lower panel are showing the view of naked eyes. (C) The time of positivity measured by real-time RT-LAMP detected with FAM channel, and the calculated sensitivity for serially diluted CHIKV RNA with or without blood were shown. The same RNA samples were used for qRT-PCR and the calculated Ct value were shown.

We have established the dried RT-LAMP system for the detection of the CHIKV genome (CHIKV-CZC-LAMP) from clinical samples, containing dried-reagents in the reaction tubes, and which were stored at ambient temperatures for prolonged periods. The CHIK-CZC-LAMP system consists of reaction tubes, reaction buffer, an isothermal incubator, and a LED LAMP reaction detector (Fig 1A). The sensitivity of the CHIKV-CZC-LAMP was evaluated using purified RNAs from CHIKV pulsed with or without 1 μl of human blood. Positive reactions were recognized as yellow fluorescence under a LED detector, and a positive reaction could also be recognized as change of sample color from violet to sky blue by the naked eye (upper column in Fig 1B). As for blood-added samples, the reaction tubes were briefly centrifuged to recognize fluorescence more clearly (lower column in Fig 1B).

The time of positivity to obtain an amplification signal by the CHIKV-CZC-LAMP was correlated with Ct values. The time required for the amplification of 50 PFU CHIKV genome was 15 to 18 min in the absence of blood, and 18 to 23 min for CHIKV genome in the presence of blood (Fig 1C). The reaction time of CHIKV genome with blood was delayed a few minutes compared to that without blood, suggesting that the delay of the reaction time may be due to inhibitory effect of the reaction by blood or more probably fluorescent masking effect by the blood color.

The sensitivity of CHIKV-CZC-LAMP using CHIKV genome at the end points of the reaction (45 minutes) was <50 PFU CHIKV per reaction, showing 100% sensitivity (95% CI: 54%-100%) at 50 PFU of CHIKV, and 33% (95% CI: 4%-78%) at 5 PFU of CHIKV. Addition of blood to the CHIKV genome had no inhibitory effect on sensitivity of the RT-LAMP, showing 100% sensitivity (95% CI: 54%-100%) at 50 PFU of CHIKV, and 50% sensitivity (95% CI:12%-88%) at 5 PFU CHIKV (Fig 1C). The sensitivity and Ct value of qRT-PCR system [25] was also determined using the same RNA templates, and was revealed to be <0.5 PFU CHIKV per reaction (Fig 1C).

### Evaluation of CHIKV-CZC-LAMP system using patient samples

The feasibility of established CHIKV-CZC-LAMP for patient sample diagnosis was validated at Flavivirus Laboratory using both RNA and serum samples of patients with chikungunya collected and kept at the Fiocruz. RNA and serum from healthy individuals (n = 4) were also tested as healthy endemic controls. All the RNA samples were tested by qRT-PCR, and the Ct values were determined (Table 1). The RNA samples and crude sera from the same patient were examined by the CHIKV-CZC-LAMP. Among 33 CHIKV qRT-PCR positive samples, 23 RNA samples and 19 serum samples were positive for CHIKV-CZC-LAMP, showing 70% (95% CI: 0.51–0.84) sensitivity for RNA samples and 58% (95% CI: 0.39–0.75) sensitivity for serum samples. No positive reaction was observed in the 4 endemic healthy control RNA and serum samples, demonstrating 100% specificity (95% CI: 0.40–1.00) (Table 1). CHIKV-CZC-LAMP-detectable RNA samples had Ct values from 12.21 to 31.1 by qRT-PCR. The results were correlated with the analytical sensitivity of the CHIKV-CZC-LAMP using RNA from CHIKV, which was 33% at 5 PFU, corresponding to 30.60 of Ct value of qRT-PCR (Fig 1C). In addition, CHIKV-CZC-LAMP showed positive reactions using crude serum from serologically positive samples which had Ct values from 12.21 to 28.43 (Table 1).

**Table 1:** RT-LAMP results using RNA and serum from chikungunya patient and endemic healthy control in Rio de Janeiro, Brazil.

ID/collection year	Days post symptom onset	qRT-PCR Ct value	RT-LAMP Template: RNA	RT-LAMP Template: serum
3080/2018	3	12.21	positive	positive
7211/2018	3	14.93	positive	positive
9327/2016	2	15.10	positive	positive
7242/2018	1	15.65	positive	positive
2926/2018	1	15.84	positive	positive
7214/2018	1	16.63	positive	positive
7502/2018	7	17.05	positive	positive
7501/2018	7	17.48	positive	positive
2285/2018	3	18.54	positive	positive
3200/2018	2	18.87	positive	positive
1294/2016	2	19.60	positive	positive
8232/2018	1	20.69	positive	positive
7358/2018	3	21.37	positive	positive
7496/2018	1	21.61	positive	positive
7209/2018	0	23.08	positive	N.D.
7497/2018	10	23.75	positive	positive
2484/2018	5	23.93	positive	positive
3083/2018	4	23.95	positive	N.D.
1268/2016	4	25.40	N.D.	positive
8811/2016	4	27.20	N.D.	N.D.
3028/2018	0	27.50	positive	positive
11555/2016	4	27.60	positive	N.D.
3201/2018	3	27.75	N.D.	N.D.
7441/2018	2	28.43	positive	positive
2285/2016	2	30.00	N.D.	N.D.
2533/2018	2	30.09	N.D.	N.D.
2011/2018	4	30.29	N.D.	N.D.
2745/2018	0	30.87	positive	N.D.
3196/2016	2	31.10	positive	N.D.
7305/2018	4	31.79	N.D.	N.D.
8350/2016	4	32.00	N.D.	N.D.
0881/2016	3	32.00	N.D.	N.D.
8831/2016	4	36.90	N.D.	N.D.
9766/2016	healthy	N.D.	N.D.	N.D.
9767/2016	healthy	N.D.	N.D.	N.D.
1364/2016	healthy	N.D.	N.D.	N.D.
1366/2016	healthy	N.D.	N.D.	N.D.

RNA and serum samples from 33 chikungunya patients and 4 endemic healthy control patients were examined by the CHIK-CZC-LAMP. As a comparison, the Ct values of clinical samples were determined using qRT-PCR. The table were arranged in ascending order of the qRT-PCR Ct value. N.D.: not detected.

### Genotyping of chikungunya virus by CHIKV-CZC-LAMP and MinION

**Fig 2:**
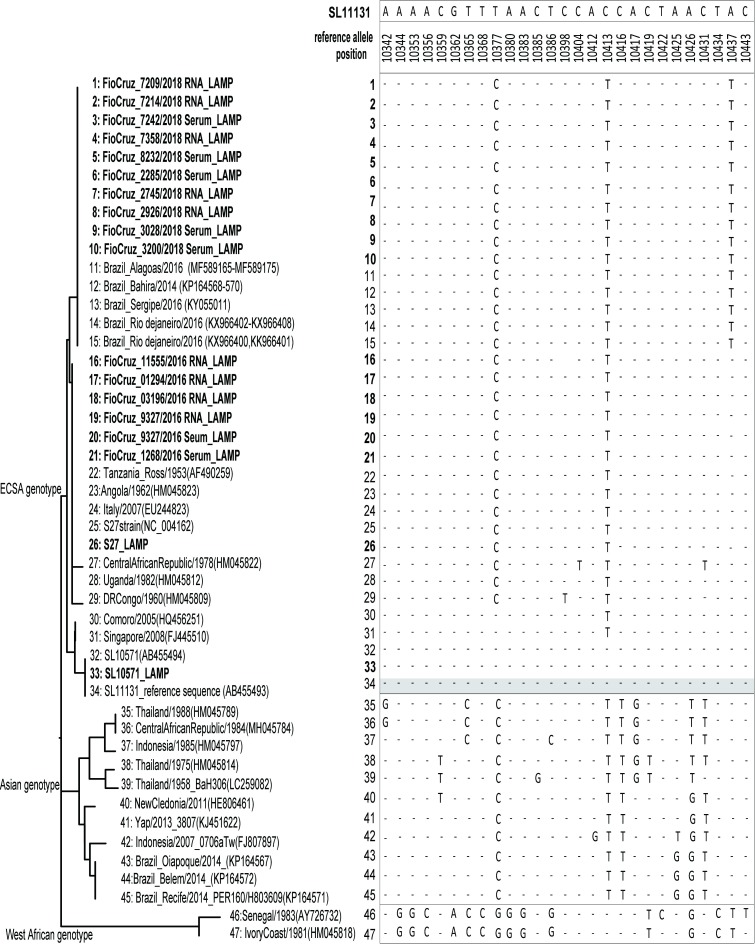
Phylogenetic tree of sequences from LAMP-Nanopore analysis. NJ tree based on 117 bp sequences determined by CHIKV-CZC-LAMP-MinION sequencing (bold) analysis, and 29 sequences from the GenBank were constructed. The nucleotides at the 27 SNPs within the amplified region were also indicated.

The amplified products by CHIKV-CZC-LAMP from clinical samples, and CHIKV-SL10571 and -S27 laboratory strains, were sequenced with MinION (Table 2). After mapping the reads to the reference sequence of CHIKV SL11131, the mapped read number of each nucleotide was retrieved, and consensus sequences were generated based on the population. The population for the major nucleotides was interpreted as a consensus of sequence although the presence of viral quasispecies could not be excluded. Because the clinical samples were sequenced with the recent version of R9.4, the average length of read and outputs were much higher in those samples than laboratory strains CHIKV-SL10571 and -S27 strains which were sequenced with the earlier version R7.0. (Table 2). Both platforms showed good coverage with no low coverage regions (>416 coverage at minimal, as shown in Table 1 and S1 Table). The lack of 3→ 5 exonuclease activity in *Bst* DNA polymerase may result in error amplification estimated to be about 1 x 10^−4^ [31]. In addition, it has been reported that sequence accuracy of the current version of the MinION R9.4 flowcell with 2D chemistry is 97% [32] which is still of course low compared to the conventional Sanger-sequence or other next generation sequencers. Nevertheless, the high sequence coverage renders enough discriminative power for single nucleotide variations (SNVs) calling with high confidence. The obtained consensus sequences were aligned, and phylogenetic trees were constructed based on 117 nucleotides of the LAMP target region (Fig 2). The sequences from two laboratory strains, CHIKV-SL10571, and African prototype CHIKV-S27 showed a 100% match with deposited sequence data, demonstrating the proof-of-concept of our analysis. For the chikungunya patient samples from Rio de Janeiro, the obtained sequences all clustered within ECSA genotype in the phylogenetic analysis (Fig 2), confirming the circulation of the ECSA genotype in Rio de Janeiro during the epidemics in 2016 and 2018.

**Table 2:** MinION sequence summary.

Sample ID	Template of LAMP	Number of reads*	Total reads bp	Average reads length	Number of sequence aligned^†^^,^	Average of major allele %^‡^	Minimal Coverage^§^
11555/2016	RNA	17,451	12,729,036	729	12,335	94.8	4,229
01294/2016	RNA	8,317	5,100,998	876	5,055	94.4	1,845
03196/2016	RNA	2,988	7,177,214	863	6,952	94.5	2,495
9327/2016	RNA	5,825	2,886,343	966	2,285	94.5	837
9327/2016	serum	2,336	2,432,949	1,042	1,567	94.2	505
1268/2016	serum	5,101	3,824,032	750	3,463	94.5	1,143
7209/2018	RNA	23,285	18,516,153	795	18,607	94.2	7,596
7214/2018	RNA	20,250	16,213,247	801	15,893	94.2	6,615
7242/2018	serum	16,463	12,769,769	776	13,689	94.3	5,327
7358/2018	RNA	9,386	7,676,569	818	8,407	94.2	3,490
8232/2018	serum	30,311	24,252,642	800	25,221	94.2	9,842
2285/2018	serum	22,375	17,944,288	802	19,093	94.3	7,739
2745/2018	RNA	14,920	12,180,109	816	12,993	94.3	5,305
2926/2018	RNA	9,874	8,086,328	819	8,288	94.3	3,480
3028/2018	serum	21,576	17,383,960	806	17,752	94.3	6,674
3200/2018	serum	31,780	25,038,530	788	25,425	94.2	1,0144
S27	RNA	5,648	2,194,824	389	4,096	94.0	1,610
SL10571	RNA	1,035	392,715	379	1,029	93.5	416

LAMP products amplified from patient RNA or serum were processed for MinION sequencer.

*Number of reads: number of the quality "passed" sequences.

^†^Number of sequence aligned: The obtained reads were blasted against reference genome SL11131 (156 bp: 10,317–10,472), and all the hit were clipped and mapped against reference again by bowtie. The number of mapped reads were shown.

^‡^Average of major allele %: The average percentage of the called dominant allele for each reference nucleotide position (F2/B2 region removed 117 bp: 10,336–10,452). The bi-allelic loci (position 10,437) was excluded from the calculations.

^§^Minimal coverage: The minimal number of reads that were aligned to reference bases (10,336–10,452).

## Discussion

The present study describes the development of a one-step, easy and “user-friendly” gene amplification assay for the rapid detection of CHIKV, named CHIKV-CZC-LAMP. The analytical sensitivity of CHIKV-CZC-LAMP was <50 PFU per reaction both from purified RNA and RNA sample pulsed with human blood. This sensitivity of the CHIKV-CZC-LAMP was not as high as the standard qRT-PCR method which showed higher sensitivity, detecting <1 PFU per reaction [25]. CHIKV-CZC-LAMP also showed less sensitivity than qRT-PCR using clinical samples. However, improvement on the sensitivity by selection of different primer sets will be expected to allow detection of relatively low viral load samples. In the current study, we used already established primer sets [24] which targeted E1 region of CHIKV genome. Other primer sets targeting the 6K-E1 regions [33] have recently been reported, which need to be comparatively validated. Also, a second set of reaction accelerating primers (stem primes) could increase the sensitivity of the amplification [34]. For the diagnosis of CHIKV infection, RNA detection methods are recommended before 6 days post symptom onset [35]. The viral titer has variation based on the individuals or the viral genotypes. It has been reported that high viral load of CHIKV (10^7^−10^9^ viral particles/ml) was detected in the patient’s blood in a recent outbreak [12], which will be easily detected in our LAMP-MinION system. After 5 days of the onset of symptom, additional serological assays are recommended, since the viral genome amount is expected to be low at this point [12, 35]. The negative results by our system in some CHIKV-infected patients who had relatively low viral load may be caused by delayed onset of symptoms after CHIKV infection. The undetectable RNA samples showed Ct value of >23.95 in qRT-PCR, which is estimated to be about 500 PFU virus per reaction, demonstrating that CHIKV-CZC-LAMP could detect only high viral titer samples in the early phase of the CHIKV infection. Therefore, we propose that our system can be combined with a serological diagnostic test. Nevertheless, the developed CHIKV-CZC-LAMP could be considered to be superior to a qRT-PCR method in terms of feasibility as there is no need for significant technical skills or expensive equipment. In addition, it has the advantages of easy transportability and low cost. This system will also be useful for diagnosis not only of chikungunya but also other established and newly emerging infections of RNA viral diseases in the field setting as a point of care test.

In Brazil, the first autochthonous cases of the Asian and ECSA genotypes were reported in 2014 in Oiapoque and Feira de Santana, respectively [9–11]. In 2016, an outbreak of chikungunya was reported in Rio de Janeiro, and ECSA genotype was reported in this epidemic [10, 16]. In this study, we decided to evaluate our CHIK-CZC-LAMP combined with MinION portable sequencer using samples from chikungunya patients in Rio de Janeiro, to determine if on-site diagnosis and on-site genotyping was feasible or not. It was demonstrated that molecular diagnosis and on-site sequencing from clinical samples in resource-limited region was possible. The sequence data obtained from the chikungunya samples revealed that those were the ECSA genotype, which was consistent with the previous report [10,16]. As a proof-of-concept, laboratory CHIKV strains (SL10571 and S27) were also sequenced and showed absolute match with the sequences deposited in GenBank. The reliability of LAMP diagnosis is also complemented by sequencing, as the LAMP method is known to cause frequent non-specific amplification induced by primer dimers [36]. We confirmed the sequence of CHIKV genome in the samples, demonstrating that the obtained sequence information gave us definitive and reliable information from epidemic clinical samples.

Distinguishing CHIKV from Dengue fever or Zika fever is also important, but it is often challenging in the clinical setting, as these viruses share the same vectors, and have similar presenting clinical symptoms. The evidence of the co-circulation of dengue virus (DENV), Zika virus (ZIKV) and CHIKV has been also reported [37,38]. Hence, multiplex or panel of diagnostics for those arbovirus infections will be also required. The RT-LAMP methods for ZIKAV and DENV has been available [39,40,41], and the MinION analysis from DENV RT-LAMP had been also been established [42]. Thus, we anticipate such multiplex assays will soon be established.

The re-emerging ECSA genotype was reported to have adapted to the *Aedes albopictus* mosquito, and to produce more virus particles in that mosquito population [43], which might result in large urban epidemics. It was also reported that ECSA genotype CHIKV infection provoked high viral load in the patient [12], suggesting that adaptive mutation in the CHIKV envelope causes high replication efficiency [17]. In addition, acquired immunity after CHIKV infection has been reported to be critical for further protection against CHIKV infection [44]. The immunity is long-lasting and suggested to be cross-reactive based on the combination of the genotypes (serotypes) [45]. Therefore, cohort studies with identification of pathogen lineages will be required for a better understanding of chikungunya epidemics. Many lineages from diverse geographical areas have potential to spread out to other geographies through travel, vectors, and reservoir animals. However, in most of the epidemic cases, sequence analysis under resource-limited conditions remains very challenging. The present study demonstrated that by combining LAMP and MinION, sequencing on site is very feasible. The blood or serum sample are directly applied to CHIK-CZC-LAMP system without RNA extraction and RT-LAMP can be performed with a battery driven portable incubator and without the need for specialized equipment. The same device can be used for MinION sample preparation, and sequencing can be done with a laptop computer. Therefore, the system is not dependent on stable electricity. The estimated cost of CZC-LAMP system was approximately one dollar per tube, which is much more affordable than RT-PCR or qRT-PCR. The MinION platform requires lower initial costs than other sequencer devices, and can read multiplexed samples, although we only used small sample size in this study. If more sample numbers are applied in one flow cell, the cost for the system will also be reduced.

In summary, a one-step, easy gene amplification assay for CHIKV genome detection was successfully developed. The assay was evaluated with RNA and serum samples from 16 CHIKV serology positive patients during recent chikungunya outbreaks in Rio de Janeiro, Brazil. In combination with MinION sequencing technology, we also identified the CHIKV genotypes with a laptop computer. The developed CHIK-CZC-LAMP diagnostics and MinION sequencing workflow will certainly contribute to future outbreak analysis in resource limited settings.

## Supporting information

S1 Table The count data and sequence obtained from MinION sequence. (XLSX)
